# Challenges to research implementation during public health emergencies: anecdote of insights and lessons learned during the COVID-19 pandemic in Gujarat, India

**DOI:** 10.3389/fpubh.2024.1417712

**Published:** 2024-07-25

**Authors:** Farjana Memon, Modou L. Jobarteh, Komal Shah, Anish Sinha, Monali Patel, Shailee Patil, Claire Heffernan, Deepak B. Saxena

**Affiliations:** ^1^Department of Epidemiology, Indian Institute of Public Health Gandhinagar, Gandhinagar, Gujarat, India; ^2^Department of Population Health, London School of Hygiene & Tropical Medicine, London, United Kingdom

**Keywords:** COVID-19, pandemic, health emergencies, health systems, lessons, insights

## Abstract

Health emergencies, including pandemics, are not new occurrences; some notable ones occurred in the past. However, the scale of the COVID-19 pandemic is unprecedented. The COVID-19 pandemic exposed the unpreparedness of national health systems in effectively managing health emergencies. During the pandemic, controlling the spread of the virus and hopes of exiting into a post-pandemic era were reliant on research to improve patient care and inform government policies. Nonetheless, research implementation during health emergencies can be challenging in low-resourced settings. This paper presents anecdotes of experiences and offers insight into ways research can be supported during health emergencies. We implemented a longitudinal study to investigate the impact of the COVID-19 pandemic, including SARS-CoV-2 infection, during pregnancy on maternal and child health outcomes. The study utilized hospital databases to recruit women who were infected and with no known SARS-CoV-2 infection during pregnancy. Mother-infant pairs in the infected and uninfected group were then followed longitudinally for 3 years. Observations, including challenges during planning, record retrieval, tracking, recruitment, and follow-up of eligible women, were reported by research staff. The challenges observed were group into three overarching themes: (a) individual factors, (b) health system challenges, and (c) research operational challenges. Some notable observations include misinformation, misconception, mistrust, underdeveloped health record systems, stigma, and hesitance. Early planning, effective communication, and community awareness can help in implementing a successful research project. Additionally, efforts to improve collaboration and co-creation between health practitioners, researchers, and the public may benefit the implementation of research projects during a health emergency.

## Introduction

The COVID-19 pandemic is probably the biggest global health emergency in recent times. What started as a respiratory disease caused by a strain of the coronavirus family quickly spread into a global pandemic, infecting, and causing the death of millions of people (1, 2). In fact, its impact was so devastating that national systems such as health, finance, food, and transport systems were brought to (near) standstill (3, 4). Health systems, in particular, had to be repurposed in many countries to meet the growing health demands of the pandemic, overwhelming service delivery, and as a result, causing disruption to other important parts of the system including maternal and child health services, cancer care and surgeries (1, 5). The disruptions, inadvertently, plays significant contributing factor in the spike in maternal death, preterm and stillbirth recorded in multiple countries in the early phase of the pandemic (1, 3–7).

In its early days, the global efforts towards controlling the spread of COVID-19 virus were based on implementation of preventative measures including handwashing, use of face mask, and public lockdown (7, 8). As the virus spreads, however, research efforts had to be intensified to inform the development and implementation of effective and safe vaccines, inform clinical management of patients, and understanding the biology and transmission dynamics of the virus (9). These research efforts have thankfully led to production of highly effective vaccines, improved clinical care of patients and supported government policies and programs. While this may sound like a resounding victory against an invading pathogen, the path to delivering a post-pandemic era was not straightforward. Barriers, obstacles, and hurdles had been to be broken, crossed, or hooped over to protect public health and wellbeing. While different countries faced different challenges, the overriding theme to describe the management of the pandemic is ‘chaotic and disruptive’. The pandemic was marked with malicious spread of misinformation, disinformation, and nationalism, contributing to distrust, hesitance, and pessimism among the public (10–12). Nevertheless, some countries were better prepared and managed the ‘chaos and disruption’ comparatively well.

In context, higher income countries contributed substantially to vaccine developments, clinical trials, and biological research, owing to their excellent research capacity, infrastructure, and funding. Low-middle income countries (LMICs) were evidently behind, largely, due to an underdeveloped (or developing, in a more optimistic term), underfunded research capacity. Despite these historical challenges, conducting research projects involving face-face interaction during the pandemic in LMICs came with additional challenges. This paper presents research staff reported experience of challenges encountered in implementing a prospective research cohort during the COVID-19 pandemic in Gujarat, India, and offers insights into ways to effectively support research project during health emergencies in resourced limited setting.

## Collection of staff perspective of the challenges encountered

Staff observations of challenges encountered in planning, implementing, and conducting a prospective research project during the COVID-19 pandemic were collected. The observations made pertain to a longitudinal study designed to investigate the impact of the pandemic, including SARS-CoV-2 infection, during pregnancy on birth outcomes, growth, and development in early childhood in Gujarat, India (13). The study recruited women who were infected with SARS-CoV-2 during pregnancy and those with no known infection. Infants born to the women in two groups (infected and non-infected groups) were followed longitudinally for over 3 years to assess their growth and cognitive development. The study has multiple strands of data collection spanning from pregnancy to early childhood, and includes extensive field visits to households of consenting women in two districts, Ahmedabad and Sabarkantha, in Gujarat: an urban and rural population, respectively. The study enrolled more than 600 mother-infant pairs. Households of consenting participants were visited at enrolment, and 6 monthly visits within a 3-year period.

As no in-depth qualitative interview questionnaires were developed to systematically collect the views of staff, three rounds of in-house group discussions were held to draw, as much as possible, the narrative of experiences of project staff regarding challenges encountered during the course of the project. The project has a total of 15 individual staff including senior scientific staff, ECR (early career research), Ph.D. students, field data collectors (enumerators) and statisticians. The research concept was introduced to the staff at the first round of in-house discussions and given time to think of the challenges encountered. At the second round of the discussions, participants (project staff) were asked their perspective of the challenges encountered during the course of the project. Participants were precisely asked to record or state the challenges they encountered, individually or collectively, during the project, and to suggest improvements that can be made to support similar research projects in similar circumstances (i.e., a health emergency) in Gujarat, India. The discussions were focused on the challenges and opportunities around the life course of implementing a research project, including:

- Planning of the project
- Obtaining institutional ethics approval
- Contacting stakeholders at healthcare institutions in Gujarat for patient information
- Contacting and enrolling eligible women
- Follow-on field visits and conducting the research

A dedicated staff was responsible for collation of responses from participants during the forum discussions. The staff manually reviewed and analyzed the interview responses to identify and extract key reoccurring themes. A report was then produced based on repeated or shared themes from the respondents. A final round of discussion was held to share the report with staff to facilitate an agreement on the outcomes. The multiple rounds of discussions were held to improve quality of responses and ensure the outcomes represent the wider views/perspective of the staff.

## Synopsis of the reported challenges and opportunities for improving research uptake

**Figure 1:**
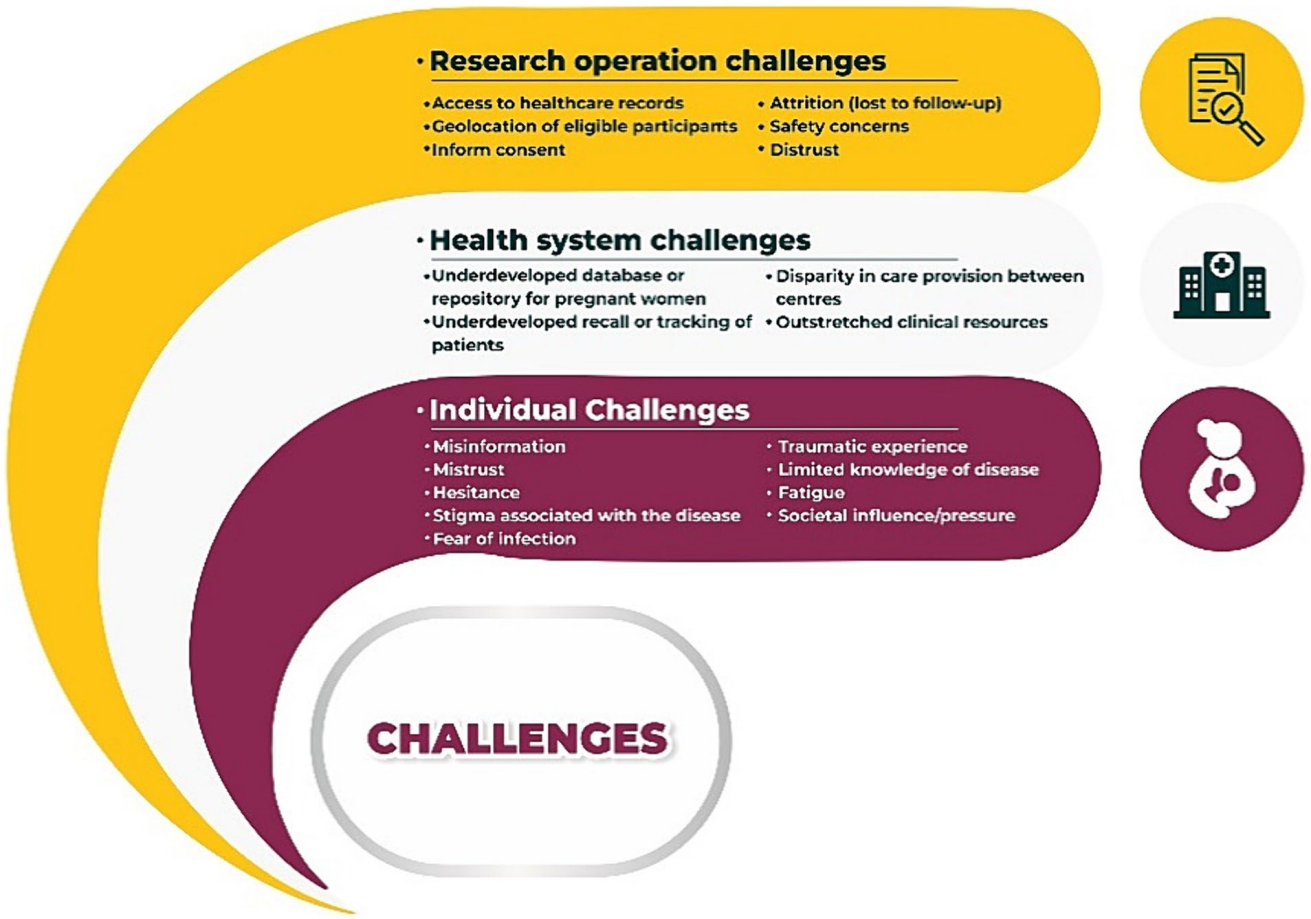
Staff reported challenges to implementing a prospective pregnancy research cohort during the COVID-19 pandemic in Gujarat, India.

**Figure 2:**
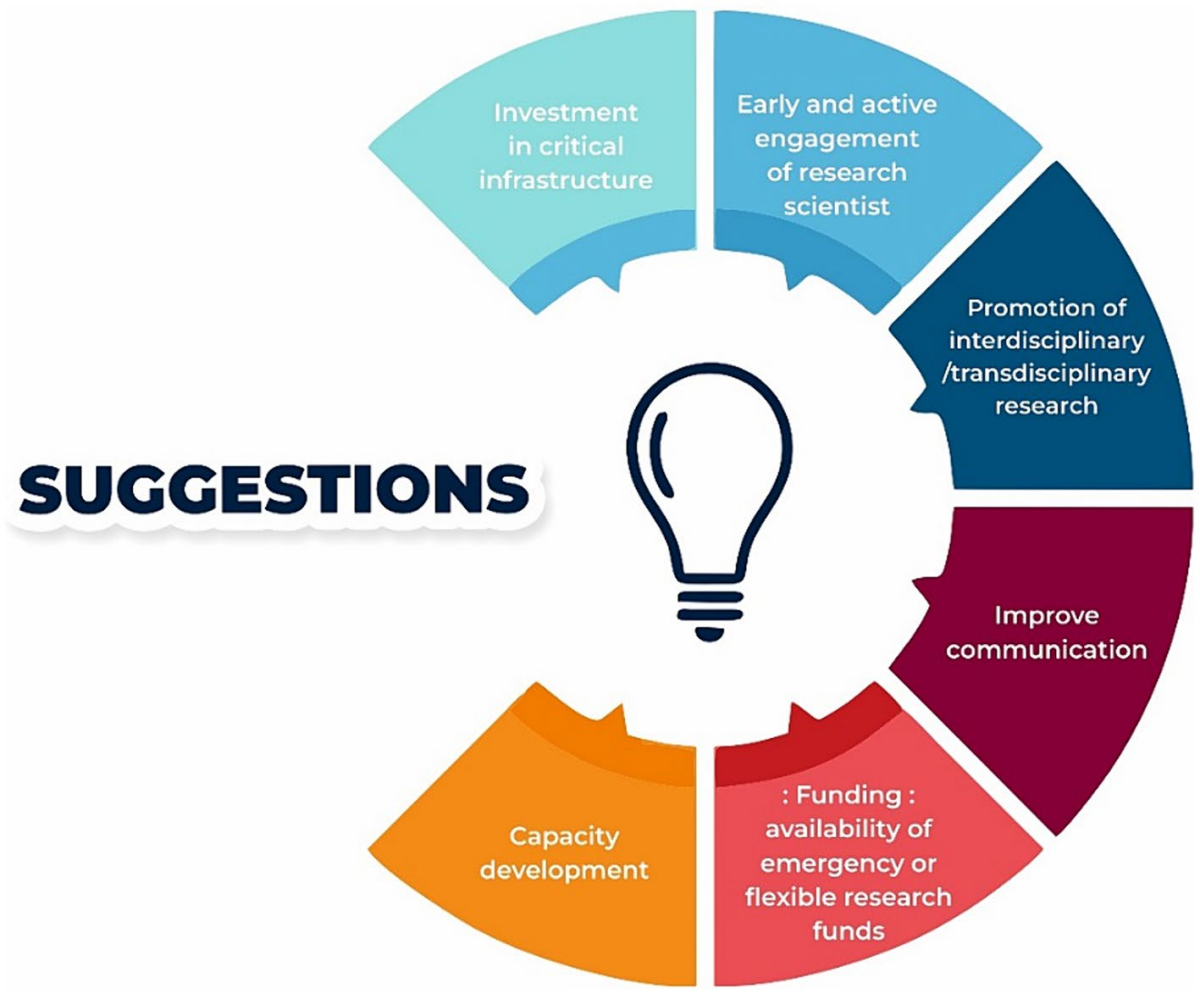
Staff suggestions of ways to support effective research implementation during a health emergency in Gujarat, India.

Staff perspectives of challenges encountered in implementing the prospective pregnancy cohort during the COVID-19 pandemic and suggestions on ways to improve implementation of future research projects during similar health emergencies in Gujarat are presented in Figures 1, 2.

The challenge encountered during the research project were grouped into three themes: individual, health system and research operational challenges. Staff reported that many of the healthcare facilities involved in the study have an underdeveloped antenatal care database or repository with minimal ability for effective recall or tracking patients. Staff unanimously reported perceptions of misinformation, mistrust, stigma, fear, traumatic experience, and societal influences among participants contacted or enrolled into the research study. Access to healthcare records of eligible participants, geolocation of participants, attrition due to lost-to-follow and safety concerns were some of common reported challenges to the research operations (Figure 1).

Research during a health emergency in this setting can be improved through a combination of investment in healthcare infrastructure (databases and registers), capacity development of staff, availability of flexible funding, improve communication and partnership between researcher, healthcare practitioners and the publics (Figure 2).

## Discussion

The COVID-19 pandemic with its high mortality, hospitalization burden and long-term health implications are avid reminders of the many emerging threats to public health, and the need for health system preparedness and resilience to counteract the threats and preserve lives and livelihoods. In effect, scientific research is critical in guiding the building of resilient national health systems and fit for purpose health policies and recommendations. But, as witnessed in the COVID-19 pandemic, research implementation during an ongoing health emergency can face various intrinsic and extrinsic challenges. In which case, swift accountability is required to learn from the various pitfalls in order to inform the strengthening of research, including its capacity and reach, during similar incidents in the future. This article reports research staff observations of challenges encountered in implementing a research project at the height of the COVID-19 pandemic in Gujarat, India, and offers suggestions on ways to improve research implementation during public health emergencies in low resourced settings.

The challenges we encountered in implementing a research project during the pandemic were many, herein grouped into three major themes: individual, health system and research operation challenges. In speaking with eligible participants contacted for enrolment into the research project, staff witnessed higher presence of misinformation, mistrust, and hesitancy among the participants. As a result, denial of the disease was common within the population. It was not uncommon for individuals contacted to participate in the study to assert a conflicting theory about the disease, with some who had positive COVID-19 test results mentioning that they did not fully trust the validity of the test, results, and existence of the disease. These beliefs posed challenges in having healthy conversations about the disease and its impact on public health. While the beliefs negatively impacted our ability to recruit and retain participants in our research project, it also has wider societal consequences. Misinformation was a primary driver of negative behavior during the COVID-19 pandemic, negatively affecting public compliance with health guidance and promoting vaccine hesitancy (14–16). Although, the sources of the misinformation are not necessarily local, their spread over popular social media sites like Facebook and WhatsApp produced ripple effects in many communities around the world, including Gujarat, India. This observation resonates with a statement released by the WHO Director-General, stating “we are not just fighting an epidemic; we are fighting an infodemic,” referring to the faster spread of COVID-19 misinformation than the virus (17). Moreover, the presence of political interference and initiatives both during and preceding the pandemic has exacerbated mistrust, particularly among religious minority groups. Along with this, the surge in nationalism witnessed during the pandemic may play a significant role in galvanizing the spread of fake news, with adverse consequences on health behaviors (18, 19). In some cases, traumatic experiences including loss of relative, neighbor, fear of contracting the virus, and not knowing much about the disease influences their decision to not participate in the study.

In working with different healthcare facilities across Gujarat, we observed that the quality of record keeping across the centers posed some challenge to the implementation of our research project. Comprehensive, consistent, and longitudinal record keeping was highlighted as a problem in many of the centers. As a result, it was sometimes difficult to obtain some important patient data required for the research project. These include disparities in record keeping practices, unstratified data, inaccurate geolocation and contact details of potential participants, impacting our ability to meet our sample size target sooner enough. Our observations align with evidence reported elsewhere underlining the universality of these challenges in healthcare record management during the pandemic (20). Furthermore, some centers were slow in fully incorporating COVID-19 surveillance and inclusion of the data into the antenatal registers. Although it could be argued that some of the antenatal data challenges predates the pandemic, the problem was exacerbated during the pandemic. Migration and reverse migration, where people move from densely populated urban centers to rural areas and then back, was common during the pandemic. The lack of adequate tracking system makes it difficult to obtain longitudinal data during follow-ups or even longer-term retainment of participants contributing to lost-to-follow up.

Therefore, to encourage and promote research implementation during health emergencies in low resourced settings, strategic investments will have to be made. Firstly, at the core of this investment is the availability of well-funded and supported infrastructure to facilitate swift implementation of research projects. This article highlights the issue of antenatal registers and database. While this is a work in progress and significant improvements have been made over the years, there is clear room for further improvement. Development of a centralized database including the conversion of paper copies of the antenatal registers to automated, user-friendly digital system will significantly improve data handling, collation, and reporting in healthcare centers. The database should have sufficient tracking and recall system to improve the tracking and care of antenatal women. Secondly, in addition to addressing the historical and ongoing problems around capacity development and retention of staff, active engagement of scientist, promotion of transdisciplinary research collaboration and availability of flexible funding will promote implementation of research projects and efficiency during health emergencies. Thirdly, while public trust in science and public health delivery may not have dwindled so much, the spread of misinformation and fake news, are barriers is facilitating a cordial public relationship. This can be mitigated through robust public awareness campaign and in some cases counter information to help debunk some damaging misinformation. Effective communication with empathy was pivotal in garnering positive responses and fostering a conducive information-sharing environment. Additionally, we found early planning and engagement of healthcare staff in the research project crucial in supporting the implementation of the project, especially for streamlining data collection and participant engagement processes.

In conclusion, this article reports challenges associated with implementing research project during the pandemic and offer suggestion of ways to improve research project during similar health emergencies. The experiences shared in this article provide valuable insights for future research on complex and sensitive topics and would enrich the planning and executing of future studies, and enhancing critical decision-making in response to emerging health challenges.

## Data availability statement

The raw data supporting the conclusions of this article will be made available by the Principal investigator, without undue reservation.

## Ethics statement

The studies involving humans were approved by Indian Institute of Public Health Gandhinagar Ethics Committee and London School of Hygiene and Tropical Medicine Research Ethics Committee. The studies were conducted in accordance with the local legislation and institutional requirements. Written informed consent for participation in this study was provided by the participants’ legal guardians/next of kin. Written informed consent was obtained from the individual(s), and minor(s)' legal guardian/next of kin, for the publication of any potentially identifiable images or data included in this article.

## Author contributions

FM: Writing – original draft, Writing – review & editing. MJ: Writing – original draft, Writing – review & editing. KS: Writing – review & editing. AS: Writing – review & editing. MP: Writing – review & editing. SP: Writing – review & editing. CH: Writing – review & editing. DS: Writing – review & editing.
